# Gluten-free diet adherence in children with screening-detected celiac disease using a prospective birth cohort study

**DOI:** 10.1371/journal.pone.0275123

**Published:** 2023-02-02

**Authors:** Pooja Mehta, Qian Li, Marisa Stahl, Ulla Uusitalo, Katri Lindfors, Martha D. Butterworth, Kalle Kurppa, Suvi Virtanen, Sibylle Koletzko, Carin Aronsson, William A. Hagopian, Marian J. Rewers, Jorma Toppari, Anette-G. Ziegler, Beena Akolkar, Jeffrey P. Krischer, Daniel Agardh, Edwin Liu, on behalf of the TEDDY Study Group

**Affiliations:** 1 Department of Pediatrics, Children’s Hospital Colorado, University of Colorado, Aurora, CO, United States of America; 2 Health Informatics Institute, University of South Florida, Tampa, FL, United States of America; 3 Health Informatics Institute, Morsani College of Medicine, University of South Florida, Tampa, FL, United States of America; 4 Celiac Disease Research Center, Tampere University, Tampere, Finland; 5 Department of Pediatrics, Tampere University Hospital, Tampere, Finland; 6 Department of Pediatrics, Seinäjoki Central Hospital, Seinäjoki, Finland; 7 National Institute for Health and Welfare, University of Helsinki, Helsinki, Finland; 8 Department of Pediatrics, Dr. von Hauner Children’s Hospital, LMU Klinikum, University of Munich, Munich, Germany; 9 Department of Pediatrics, University of Warmia and Mazury, Olsztyn, Poland; 10 Department of Clinical Sciences, Lund University, Malmö, Sweden; 11 Pacific Northwest Research Institute, Seattle, WA, United States of America; 12 Barbara Davis Center for Childhood Diabetes, University of Colorado, Aurora, CO, United States of America; 13 Department of Pediatrics, Turku University Hospital, Turku, Finland; 14 Institute of Biomedicine, Research Centre for Integrative Physiology and Pharmacology, University of Turku, Turku, Finland; 15 Institute of Diabetes Research, Helmholtz Zentrum München, Klinikum rechts der Isar, Technische Universität München, Forschergruppe Diabetes e.V., Neuherberg, Germany; 16 National Institute of Diabetes & Digestive & Kidney Diseases, Bethesda, MD, United States of America; Liv Hospital Gaziantep, TURKEY

## Abstract

**Background:**

Celiac disease has an increasing incidence worldwide and is treated with lifelong adherence to a gluten-free diet. We aimed to describe gluten-free diet adherence rates in children with screening-identified celiac disease, determine adherence-related factors, and compare adherence to food records in a multinational prospective birth cohort study.

**Methods:**

Children in The Environmental Determinants of Diabetes in the Young study with celiac disease were included. Subjects had at least annual measurement of adherence (parent-report) and completed 3-day food records. Descriptive statistics, t-tests, Kruskal-Wallis tests and multivariable logistic and linear regression were employed.

**Results:**

Two hundred ninety (73%) and 199 (67%) of subjects were always adherent to a gluten-free diet at 2 and 5 years post celiac disease diagnosis respectively. The percentage of children with variable adherence increased from 1% at 2 years to 15% at 5 years. Children with a first-degree relative with celiac disease were more likely to be adherent to the gluten-free diet. Gluten intake on food records could not differentiate adherent from nonadherent subjects. Adherent children from the United States had more gluten intake based on food records than European children (P < .001 and P = .007 at 2 and 5 years respectively).

**Conclusion:**

Approximately three-quarters of children with screening-identified celiac disease remain strictly adherent to a gluten-free diet over time. There are no identifiable features associated with adherence aside from having a first-degree relative with celiac disease. Despite good parent-reported adherence, children from the United States have more gluten intake when assessed by food records. Studies on markers of gluten-free diet adherence, sources of gluten exposure (particularly in the United States), and effects of adherence on mucosal healing are needed.

## Introduction

Celiac disease (CD) affects up to 1.4% of the world’s population [1] has a rising incidence and prevalence, particularly in Europe [2, 3], and is one of the most common childhood gastrointestinal diseases [4]. While there are limited studies in non-Western countries, the incidence of CD is likely increasing worldwide [5–7]. A strict gluten-free diet (GFD) is the only treatment for CD [8, 9], yet there are few longitudinal studies on adherence to a GFD and no international studies comparing adherence. Because inadequately treated CD is associated with growth stunting [10], nutritional deficiencies [11], neurologic and psychiatric disorders [12, 13], and impaired quality of life [14–16], understanding GFD adherence is critical in the care of CD patients.

There is currently no universal method of assessing adherence to a GFD. Gastroenterologists frequently use a combination of self and parent-reported adherence, presence of symptoms, assessment by a dietitian (if available), or serological markers. While numerous gastrointestinal societies have recommended routine assessment of GFD adherence [8, 17, 18], no standardized method of assessment exists. Moreover, children with CD are frequently lost to follow-up [19, 20] and those that are lost to follow-up are suspected to be more nonadherent [21, 22]. The aims of this study are to measure longitudinal rates of parent-reported adherence in children with screening-identified CD, determine factors related to adherence, and compare parent-reported adherence to food records in a multinational cohort of children with CD.

## Materials and methods

### Study population

**Fig 1:**
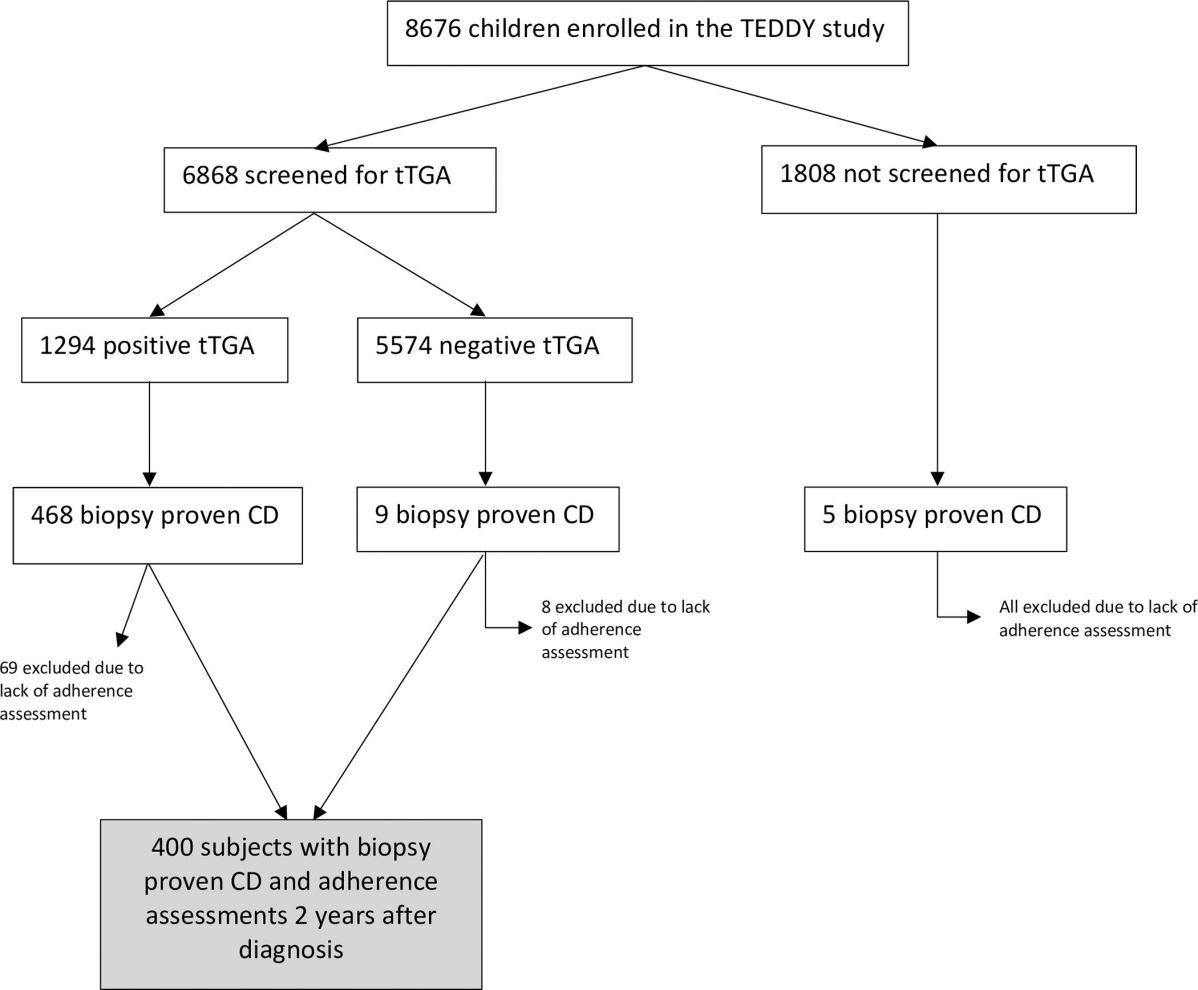
Study population. Inclusion and exclusion criteria for the current TEDDY study. Abbreviations: Celiac disease (CD); The Environmental Determinants of Diabetes in the Young (TEDDY); tissue transglutaminase autoantibodies (tTGA).

The Environmental Determinants of Diabetes in the Young (TEDDY) study is a multinational prospective observational birth cohort study that aimed to identify environmental factors associated with type 1 diabetes and CD in children at genetic risk. Subjects in the TEDDY study were followed from birth until the age of 15 years at 1 of 6 clinical research centers located in both the United States (Colorado, Georgia, and Washington) and Europe (Finland, Germany, and Sweden). Between September 2004 and February 2010, 424,788 newborns were screened with 21,589 having 1 of 9 targeted HLA genotypes. Of the infants carrying a permissive HLA genotype, 8,676 were enrolled in the prospective follow-up. Details of the study design, eligibility, and methods have been published previously [23–26]. The study was approved by local Institutional Review Boards and is monitored by an External Advisory Board formed by the National Institutes of Health. For the current study, subjects were included if they had biopsy-proven CD, were followed by the study for at least 2 years after CD diagnosis, and completed annual adherence questionnaires for at least 2 years after CD diagnosis. A detailed flowchart depicting inclusion and exclusion criteria is depicted in Fig 1.

### CD diagnosis

Serum samples were obtained from all children enrolled in TEDDY every 3 months until the age of 4 years and every 6 months thereafter. Annual screening for CD with serum tissue transglutaminase autoantibodies (tTGA) started at 2 years of age. If a child tested positive at 2 years, earlier blood samples were also analyzed. Guardians of children with a positive test result of tTGA were informed, and if tTGA remained positive on the next sample, were advised to consult a pediatric gastroenterologist for additional evaluation of CD. Subjects were defined as having CD if they had an intestinal biopsy with Marsh score of 2 or greater.

### Questionnaires and adherence definition

A specific questionnaire evaluating children for signs and symptoms associated with CD (abdominal discomfort, anemia, chronic constipation, dental enamel defects, fatigue, frequent loose stools, irritability, neurologic symptoms, vomiting, skin irritation, and poor growth) was obtained every 6 months until the age of 2 and annually thereafter. A GFD form was administered to all subjects diagnosed with CD at their next follow-up visit and annually thereafter. Subjects were considered to be “adherent” if parents reported their child ate gluten “never” or “less than once per month” in each annually-submitted questionnaire form at every follow-up until 2 years or 5 years from the diagnosis of CD. Subjects were considered to be “nonadherent” if parents reported they ate gluten “once per month,” “several times per month,” “several times per week,” or “nearly every day” in any questionnaire form during follow-up of 2 years or 5 years post the diagnosis of CD.

### Dietary assessment

The dietary assessment method used in this study has been described in detail in other publications [27, 28]. Gluten intake was estimated from 3-day food records (2 weekdays and 1 weekend day). Primary caretakers were trained on how to keep the food records during their 3-month visit. Food records were completed every 3 months for the first year of life and then biannually thereafter until 10 years of age. A harmonized food-grouping system was used to facilitate a comparison and quantification of food consumption between individual countries [29]. Mean gluten intake (grams/day) was calculated from total gluten-containing flours (wheat, rye, and barley) reported during the 3-day recording period. Protein content was obtained from the daily intake of gluten-containing flours and converted to amount of gluten using a conversion factor of 0.8 (the gluten content in wheat protein). Data regarding gluten consumption was included in this study if food records were submitted within 2–3 years after the diagnosis of CD (for the 2-year analysis) and 5–6 years after the diagnosis of CD (for the 5-year analysis).

### Statistical analysis

Descriptive statistics were used to determine the percentage of adherent and nonadherent subjects at 2 and 5 years post diagnosis of CD. Quantitative comparison of gluten intake was then performed using t-tests and Kruskal-Wallis tests. Multivariate logistic regression was used to confirm the association between parent-reported adherence with gluten intake, adjusting for country and the duration of time between measurements of parent-reported adherence and gluten intake.

## Results

### Parent-reported adherence

**Fig 2:**
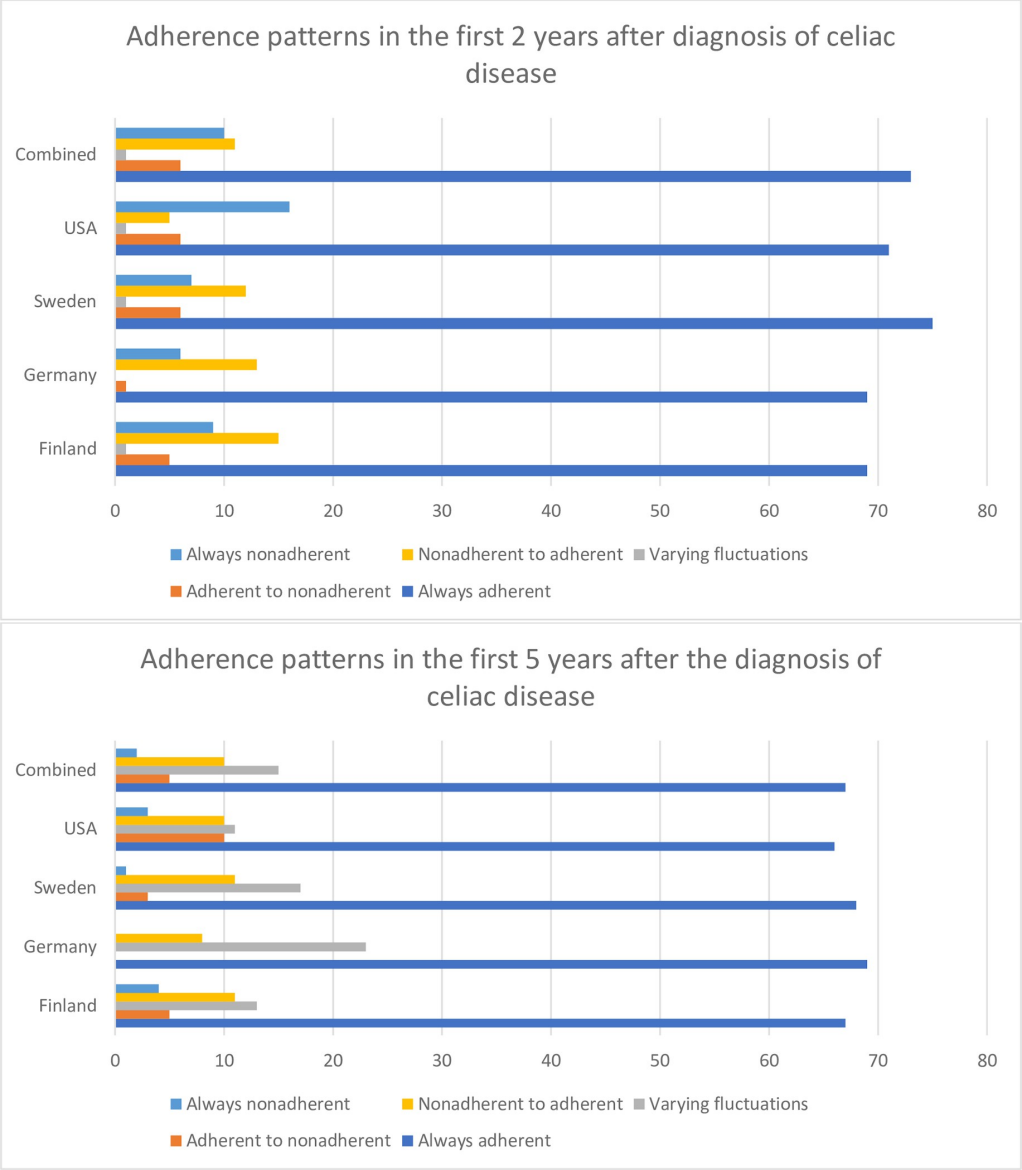
Country variations in adherence. Gluten-free diet adherence patterns stratified by country at 2 and 5 years post the diagnosis of celiac disease.

Parent/guardian-reported adherence to GFD was available for 400 subjects at 2 years post diagnosis of CD and 296 subjects at 5 years post diagnosis of CD (Fig 2). The average number of forms submitted per subject was 1.87 (at 2 years) and 4.47 (at 5 years). The majority of subjects were always adherent to a GFD at 2 and 5 years after the diagnosis of CD (73% and 67% respectively). Ten percent of subjects were always nonadherent at 2 years post diagnosis of CD while only 3% of subjects were always nonadherent at 5 years post diagnosis. One percent of subjects had with variable adherence at 2 years while 15% had variable adherence at 5 years. At 2 years post CD diagnosis, adherent subjects did not differ from nonadherent subjects with regards to sex, country of origin, community type, age at diagnosis, age at form submissions, comorbidities, and parental characteristics (Table 1). Adherent subjects were more likely to have a first degree relative with CD (21% versus 13%, *P* = .04 at 2 years and 24% versus 13%, *P* = .04 at 5 years). Nonadherent subjects were more likely to endorse gastrointestinal symptoms at 2 years only (52% versus 36%, *P* = .03).

**Table 1:** Subject characteristics. Subject characteristics at 2 and 5 years post the diagnosis of celiac disease stratified by self-reported gluten-free diet adherence.

	2 years post CD diagnosis			5 years post CD diagnosis		
Characteristic	Adherent (N = 290)	Nonadherent (N = 110)	p-value	Adherent (N = 199)	Nonadherent (N = 97)	p-value
Sex (%)			0.27			0.23
Female	187 (65)	65 (59)		132 (66)	58 (60)	
Male	103 (36)	45 (41)		67 (34)	39 (40)	
Country (%)			0.79			0.98
Finland	51 (18)	23 (21)		37 (19)	18 (19)	
Germany	11 (4)	5 (5)		9 (5)	4 (4)	
Sweden	148 (51)	50 (45)		101 (51)	48 (49)	
United States	80 (28)	32 (29)		52 (26)	27 (28)	
Location (%)			0.61			0.54
Big city	30 (10)	16 (15)		25 (13)	17 (18)	
Suburb	110 (38)	36 (33)		25 (13)	15 (15)	
Small city/village	113 (39)	44 (40)		76 (38)	35 (36)	
Rural area	34 (12)	14 (13)		71 (37)	30 (31)	
Mean child age at diagnosis in months (±SD)	55.5 (±23.5)	60.4 (±23.5)	0.22	47.9 (±18.5)	48.6 (±18.6)	0.99
Mean child age at first form submission (±SD)	62.7 (±23.2)	65.2 (±22.9)	0.49	56.7 (±18.2)	55.0 (±18.7)	0.52
Mean maternal age at diagnosis in years (±SD)	35.9 (±4.9)	35.7 (±5.3)	0.49	35.3 (±4.9)	34.7 (±4.9)	0.28
Mean paternal age at diagnosis in years (±SD)	38.8 (±5.4)	38.8 (±8.0)	0.96	37.8 (±5.7)	38.8 (±7.2)	0.48
Highest degree of maternal education (%)			0.10			0.17
Primary education–some trade school	51 (18)	22 (20)		34 (17)	18 (19)	
Graduated trade school or some college	49 (17)	26 (24)		29 (15)	22 (23)	
Graduated college or higher degree	188 (65)	58 (52)		56 (58)	134 (67)	
Missing Data	2 (1)	4 (4)		2 (1)	1 (1)	
Highest degree of paternal education			0.33			0.27
Primary education–some trade school	76 (26)	35 (32)		49 (25)	30 (31)	
Graduated trade school or some college	68 (24)	21 (19)		48 (24)	17 (18)	
Graduated college or higher degree	142 (49)	47 (43)		99 (50)	46 (47)	
Missing Data	4 (1)	7 (6)		3 (2)	4 (4)	
Comorbid Type 1 DM (%)	6 (2)	5 (5)	0.16	2 (1)	3 (3)	0.24
First degree relative with celiac disease (%)	60 (21)	14 (13)	**0.04**	47 (24)	13 (13)	**0.04**
Presence of GI symptoms (%)	104 (36)	57 (52)	**0.03**	74 (37)	39 (40)	1
Presence of non-GI symptoms (%)	10 (3)	3 (3)	1	6 (3)	3 (3)	1

### Association of adherence with food records

**Fig 3:**
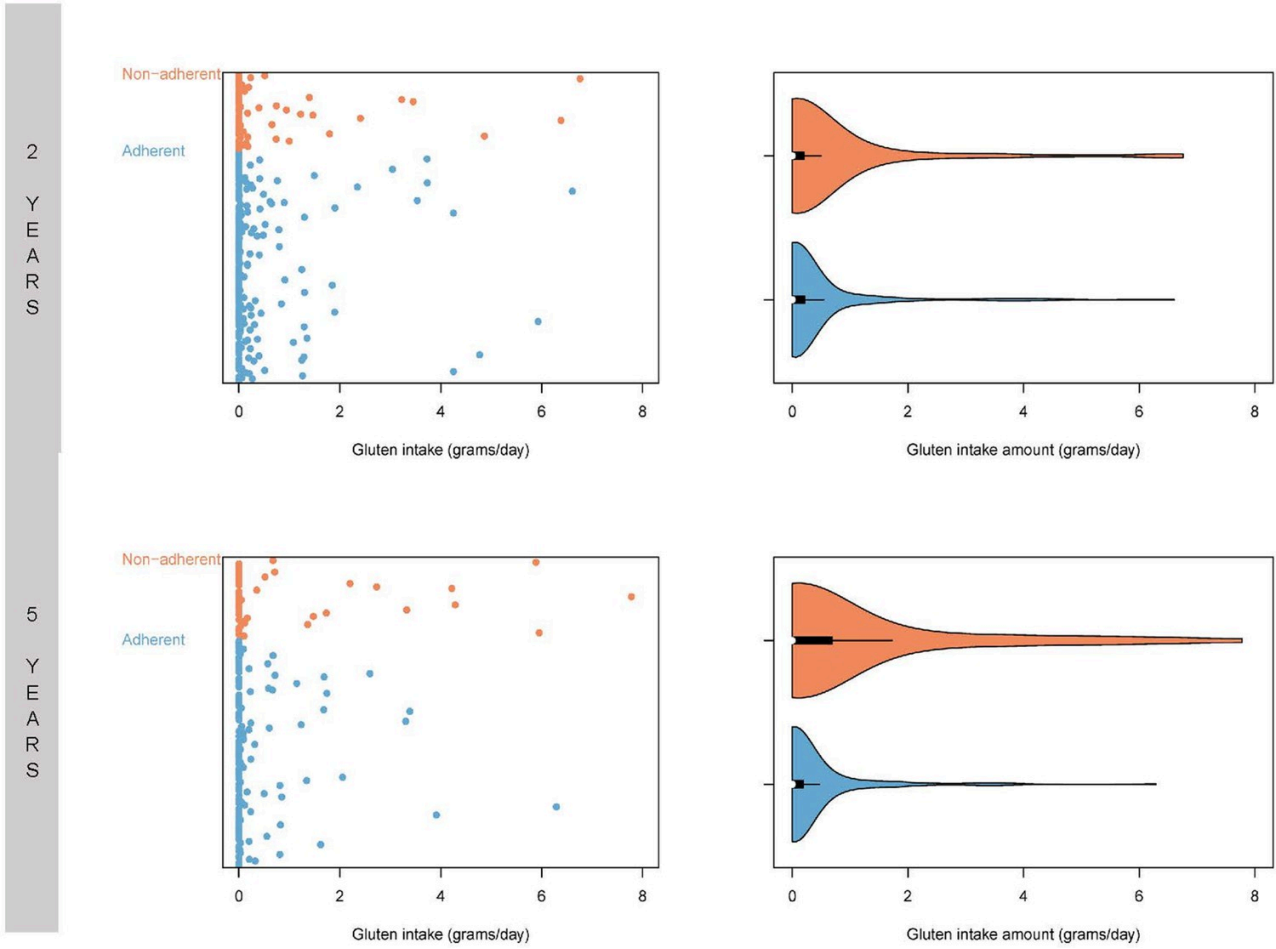
Gluten intake. Mean gluten-intake based on 3-day food records in subjects adherent and nonadherent to a gluten-free diet at 2 and 5 years post the diagnosis of celiac disease. One slice of white bread is roughly equivalent to 2 grams of gluten [30].

Fig 3 depicts distributions of gluten intake based on 3-day food records at 2 and 5 years post CD diagnosis. There was a skewed distribution of gluten intake. Nonadherent subjects had higher gluten intake using linear regression at 2 years (P < .001) and 5 years (P < .001). At 2 years post CD diagnosis, food records were available for 294 subjects. The 222 adherent subjects had a mean gluten intake of 0.36 ± 0.95 g/day and the 72 nonadherent subjects had a mean intake of 0.55 ±1.35 g/day with median intake of 0.00 (IQR 0.00, 0.22) and 0.00 (IQR 0.00, 0.20) respectively. At 5 years post CD diagnosis, food records were available for 185 subjects. The 138 adherent subjects had a mean gluten intake of 0.32 ± 0.84 g/day and the 47 nonadherent subjects had a higher mean intake of 0.93 ±1.85 g/day with median intake of 0.00 (IQR 0.00, 0.19) and 0.00 (IQR 0.00, 0.69) respectively.

### Variations by country

Mean gluten intake among countries differed in both adherent and nonadherent subjects at 2 years post CD diagnosis (P < .001 and *P* < .001 respectively) with subjects from the US having higher gluten intake (Table 2). This persisted after removing Germany from the analysis due to small sample size (*P* < .001 and *P* = .004 respectively). At 5 years post diagnosis of CD, there were significant differences in gluten intake among countries in adherent subjects (*P* = .007) with subjects from the US having higher gluten intake (Table 2). This was not noted in nonadherent subjects (*P* = .19). This persisted if Germany was removed from the analysis due to small sample size (*P* = .03 for adherent subjects and *P* = .16 in nonadherent subjects).

**Table 2:** Country level gluten-intake. Gluten-intake based on 3-day food records, stratified by country and parent-reported adherence.

2 years post diagnosis of celiac disease			
	Country	Mean gluten intake in g/day (±SD)	Median gluten intake in g/day (IQR)
Adherent	Finland (N = 27)	0.13 (±0.29)	0.00 (0.00, 0.11)
	Germany (N = 1)	2.27 (±2.83)	1.30 (0.08, 3.91)
	Sweden (N = 121)	0.26 (±0.77)	0.00 (0.00, 0.09)
	United States (N = 67)	0.42 (±0.87)	0.03 (0.00, 0.36)
Nonadherent	Finland (N = 9)	0.38 (±1.07	0.00 (0.00, 0.00)
	Germany (N = 7)	0.65 (NA)	0.65 (0.65, 0.65)
	Sweden (N = 38)	0.16 (±0.80)	0.00 (0.00, 0.00)
	United States (N = 24)	1.22 (±1.87)	0.45 (0.10, 1.41)
5 years post diagnosis of celiac disease			
	Country	Mean gluten intake in g/day (±SD)	Median gluten intake in g/day (IQR)
Adherent	Finland (N = 15)	0.02 (0.05)	0.00 (0.00, 0.00)
	Germany (N = 4)	2.75 (2.90)	2.36 (0.61, 4.51)
	Sweden (N = 81)	0.21 (0.46)	0.00 (0.00, 0.11)
	United States (N = 38)	0.41 (0.87)	0.00 (0.00, 0.22)
Nonadherent	Finland (N = 6)	0.12 (0.20)	0.04 (0.01, 0.09)
	Germany (N = 1)	1.36 (NA)	1.36 (1.36, 1.36)
	Sweden (N = 25)	0.68 (1.45)	0.00 (0.00, 0.35)
	United States (N = 15)	1.65 (2.59)	0.12 (0.00, 2.85)

## Discussion

Lifelong adherence to a GFD is the only available treatment for CD yet little is known regarding long-term dietary patterns of people with CD. In this study, we aimed to describe long-term adherence rates in children with screening-detected CD, explore factors related to adherence, and determine the association between parent-reported adherence and gluten intake based on food records. We found that nearly three-quarters of children with screening-detected CD reported maintaining strict adherence to a GFD, clinical characteristics did not distinguish adherent from nonadherent subjects, and children from the US had higher gluten intake based on 3-day food records.

### Adherence rates

Adherence to a GFD varies between 23% to 98% depending on the definition of adherence used and method of assessment [14] with comparable adherence rates found in screening detected CD patients [31, 32]. GFD adherence in screening detected patients has been a source of controversy with some arguing that universal screening for CD may lead to poorer adherence rates [33–36]. In this study, we found relatively high rates of long-term GFD adherence with only one-quarter and one-third of subjects reporting any major lapses in adherence at 2 and 5 years post the diagnosis of CD. In fact, our findings support previous research showing that earlier childhood diagnosis may in fact lead to improved GFD adherence [37, 38]. Children with a first-degree family member with CD were more likely to be adherent, perhaps due to easily accessible gluten-free foods at home. On the contrary, nonadherent subjects were more likely to report gastrointestinal symptoms which may be reflective of ongoing gluten exposure. Finally, we noted that the percentage of children with variable adherence rates increased from 1% at 2 years to 15% at 5 years. There evidence suggesting that dietary habits established in childhood will persist into to adulthood [39, 40], and that lifestyle patterns in childhood are also persistent [41]. Although 5 years is a relatively short time in the lifespan of a child, we are hopeful that the 5 year adherence data in this study may reflect eating habits and lifestyle choices such as reading labels and eating out, and thus may be representative of future adherence patterns in children. While there is limited data on longitudinal adherence rates in celiac disease specifically, it is important to note that studies examining adherence to healthy diets in weight loss have found increasing difficulty in sustained adherence [42]. Because pediatric patients with celiac disease are frequently lost to follow up [43], and those that are lost to follow have poorer GFD adherence [21, 22], our findings reinforce that not only is regular follow up throughout childhood and adolescence is needed but also that there should be continued assessment of evolving barriers to GFD adherence.

### Gluten exposure based on food records

Although nonadherent subjects had more gluten intake than adherent subjects, gluten intake was minimal in most individuals based on median values. The data once again was skewed (Fig 3) with some individuals taking 6 to 7 grams of gluten per day (approximately 3–3.5 slices of white bread per day) [30]. This may be due to intentional intake or an inadequate understanding of the diet [44]. While our study did not measure cross-contamination, the recently published DOGGIE BAG study showed that unintentional gluten intake is common and frequently measured noninvasive measures of gluten intake such as tTGA levels do not correlate with gluten-exposure in individuals with self-reported strict adherence [45]. Prior literature has also shown that self-report or other standardized measures of adherence do not always correlate well with histologic healing [46, 47]. Thus our findings in conjunction with the literature suggest that current adherence assessments and disease monitoring guidelines for children inadequately assess gluten exposure and disease activity.

### Country variations in gluten exposure

While no country level differences in reported adherence rates were noted, subjects from the US had significantly more gluten intake from food records. This may be due to several reasons. First, visits with a dietitian are not universally covered by insurances in the US. While the National Institute of Health Consensus on CD recommends consultation from a skilled dietitian [48], studies have shown that many patients with CD have not seen a dietitian or have only seen a dietitian once [49, 50]. Second, GFD foods are more costly than their counterparts [51] and countries have varying approaches to allocating resources to people with CD [52] which may affect people’s ability to buy gluten-free foods. For example, some children in Sweden are provided a $5,000 stipend to help offset food-associated costs of CD [53]. Third, European countries enacted gluten-free labeling laws earlier than the United States [54, 55]. Finally, cultural differences in eating habits such as food content of a traditional diet, frequency of dining out [56] and reliance on processed foods [57] may play a role.

There are several limitations to this study. All subjects in this study are considered to have screening-detected celiac disease. While it is possible that some tTGA positive children would have developed symptoms and likely be diagnosed shortly after seroconversion, prior work has shown that over 50% of children with screening detected celiac disease have symptoms [31] and that severity of symptoms prior to diagnosis do not affect adherence [58]. Twenty-six percent of subjects dropped out of the study between 2 and 5 years after diagnosis. This is common in prospective cohort studies and may be due to subject factors (such moving etc.) or due to the burden placed on subjects in the TEDDY study (frequent blood draws, questionnaires, and research visits) [59]. While there is potential to have a biased sample due to this, this is still the largest prospective study measuring adherence to a GFD in screening-identified children with CD and the only study to directly compare intake across countries. Second, the results of this study cannot be generalized to all children with comorbid CD and type 1 diabetes. Subjects in the TEDDY study are withdrawn from TEDDY when they develop diabetes. Thus, the subjects in our study with diabetes developed it after CD onset. Because type 1 diabetes usually precedes the diagnosis of CD [60, 61], our population is not representative of the typical population of children with comorbid diabetes and CD. Third, we did not quantify and compare tTGA levels of all subjects. All TEDDY studies use Bristol tTGA RIA levels which includes both tissue transglutaminase IgA and IgG. RIA has been shown to remain positive in over 50% of children 5 years after initiating a GFD [62] and levels can remain high even in treated children with normal intestinal mucosa [63]. Furthermore, with the exception of IgA deficiency, IgG autoantibodies are not routinely recommended in the diagnosis and monitoring of celiac disease [18]. Because negative serology is not correlated with adherence assessment by a registered dietitian [64], symptomatology [65], or indicative of mucosal healing [18], we do not feel this detracts from the study. The 3-day food records that were used to determine the gluten content of foods were based on parent-report. Although subjects were trained on these and were advised to report 2 weekdays and 1 weekend, we cannot be sure they are representative of normal eating patterns. Finally, we relied on parent report for adherence assessments. While it may not be an accurate reflection of child intake, this is what is commonly done in clinical practice, no standardized adherence measure exists for the pediatric population, and validated adult indices are not applicable internationally.

## Conclusions

In this large multinational prospective cohort study of screening-identified children with CD, we found that 73% and 67% of subjects reported strict adherence to a GFD at 2 and 5 years after the diagnosis of CD, the proportion of subjects with variable adherence increased over time, and that there were no distinguishing features of adherent and nonadherent subjects aside from having a first degree family member with CD. We also found that both adherent and nonadherent subjects had portions of subjects eating large quantities of gluten, suggesting that better markers of adherence are needed. Determination of sources of gluten exposure, particularly in the US, is warranted. Finally, future studies regarding the effects of adherence on mucosal healing and diseases outcomes are needed.
